# Genotype-Phenotype Correlation Insights Through Molecular Modeling Analysis in a Patient with Loeys-Dietz Syndrome

**DOI:** 10.3390/genes16040357

**Published:** 2025-03-21

**Authors:** Galateia Stathori, Eleni Koniari, Dimitrios Vlachakis, Eleni Papanikolaou, George P. Chrousos, Christos Yapijakis

**Affiliations:** 1 University Research Institute of Maternal and Child Health and Precision Medicine, School of Medicine, National Kapodistrian University of Athens, 115 27 Athens, Greecehelenia8@yahoo.it (E.K.); dimvl@aua.gr (D.V.); chrousge@med.uoa.gr (G.P.C.); 2 Clinical and Translational Research Endocrine Unit, School of Medicine, National Kapodistrian University of Athens, 115 28 Athens, Greece; papanikolaou.el77@gmail.com; 3 Unit of Orofacial Genetics, 1st Department of Pediatrics, School of Medicine, National Kapodistrian University of Athens, “Aghia Sophia” Children’s Hospital, 115 27 Athens, Greece; 4 Laboratory of Genetics, Department of Biotechnology, School of Applied Biology and Biotechnology, Agricultural University of Athens, 118 55 Athens, Greece

**Keywords:** Loeys-Dietz syndrome, transforming growth factor β, TGF-β2 mutations, marfanoid habitus, aortic aneurysms, vascular tortuosity, craniofacial anomalies, skeletal anomalies

## Abstract

Background: Pathogenic variants within the gene encoding transforming growth factor β (TGF-β) are responsible for Loeys-Dietz syndrome (LDS), a heritable thoracic aortic disease sharing clinical features with Marfan syndrome, including craniofacial and skeletal abnormalities as well as aortic root aneurysms and dissections. In contrast to Marfan syndrome patients, who rarely develop aneurysms or dissections beyond the aortic root, LDS patients frequently exhibit vessel aneurysms in locations other than the aortic root. Here, we report the case of a 61-year-old patient who initially presented with marfanoid characteristics and an aortic root aneurysm and was presumed to have Marfan syndrome two decades ago. Later, the patient developed an abdominal aorta aneurysm, necessitating endovascular repair and stent placement. That fact raised doubts regarding the initial diagnosis of Marfan syndrome, and we decided to investigate the genetic cause of the disorder. Methods: Genetic testing was performed using WES analysis and Sanger sequencing. Results: The genetic analysis detected a de novo heterozygous pathogenic variant c.896G>A in exon 5 of the *TGFB2* gene, resulting in the amino acid substitution p. Arg299Gln that has devastating destabilizing structural effects on 3D folding of the protein, as demonstrated by the molecular modeling study we performed. This variant is pathogenic for LDS type 4, partially consistent with the patient’s clinical presentation. Conclusions: Our case emphasizes the significance of precise clinical assessment and genetic verification in patients exhibiting marfanoid characteristics. Furthermore, our findings contribute to the understanding of the diverse clinical spectrum associated with this specific pathogenic variant of *TGFB2*, underscoring the importance of detailed clinical assessment in expanding knowledge of genotype-phenotype correlations. Accurate diagnosis is crucial for tailored and appropriate management of individuals with heritable thoracic aortic diseases.

## 1. Introduction

Initially described in 2005, Loeys-Dietz syndrome (LDS) is a rare hereditary connective tissue disorder characterized by its dominant autosomal inheritance pattern, affecting multiple body systems [1]. Its most common manifestations are aortic aneurysms, vascular tortuosity with aneurysm formation in vessels other than the aorta, hypertelorism, bifid uvula, and cleft palate [2]. Nonetheless, individuals with LDS can exhibit a wide array of symptoms. Although LDS shares clinical similarities with Marfan syndrome, primarily in terms of vascular, craniofacial, skeletal, and cutaneous features, the two syndromes have distinct origins and some unique clinical attributes that facilitate their differentiation [2].

The initial classification of LDS relied on the presence or absence of craniofacial abnormalities. However, as molecular techniques have advanced, the contemporary categorization of LDS is now primarily determined by the identification of the specific gene pathogenic variant responsible for the syndrome [3]. Thus far, there have been five officially recognized subtypes of LDS: LDS1 is associated with pathogenic variants in the gene encoding transforming growth factor β receptor 1 (TGFBR1); LDS2 is associated with pathogenic variants in the gene encoding transforming growth factor β receptor 2 (TGFBR2); LDS3 is related to pathogenic variants in the gene encoding “suppressor of mothers against decapentaplegic homolog 3” (SMAD); LDS4 is related to pathogenic variants in the gene encoding the transforming growth factor β2 (TGFB2) ligand; and LDS5 is related to pathogenic variants in the gene encoding the transforming growth factor β3 (TGFB3) ligand [3]. Importantly, mutations in SMAD2 have also been linked to clinical characteristics of LDS such as arterial aneurysms [4,5]. These pathogenic variants have recently been classified as part of LDS type 6.

Among the Loeys-Dietz syndrome types, LDS4 appears to have a stronger resemblance to Marfan syndrome. In the existing literature, there are few case reports and cohort studies related to LDS type 4 [3,6,7,8,9,10]. A recent systematic review has revealed that individuals with LDS type 4 commonly display a high prevalence of aortic aneurysms (with a prevalence ranging from 22% to 100%), joint laxity, pectus deformities, and scoliosis [3]. Furthermore, frequently observed characteristics include arachnodactyly, a high-arched palate, pes planus, and the presence of umbilical or inguinal hernias. Aortic dissection was found to occur in approximately 9% to 11% of the cases [3].

Individuals with both Marfan syndrome and LDS4 typically exhibit aortic aneurysms, arachnodactyly, scoliosis, pectus deformities, and skin striae [7]. In contrast, arterial tortuosity, hypertelorism, bifid uvula, club feet, and the characteristic thin skin with easy bruising are exclusive to individuals with LDS4. Meanwhile, the presence of ectopia lentis has been suggested as a distinguishing hallmark of Marfan syndrome in the context of connective tissue disorders [7,11]. Patients with Marfan syndrome typically display distinctive aortic root aneurysms (with a Z-score of ≥2 when adjusted for age and body size). In contrast, LDS4 patients exhibit diffuse aortic and other arterial aneurysms, although the latter is more frequently seen in LDS types 1 and 2 [12]. It is worth noting, however, that Marfan patients rarely present with dissections of the descending aorta (reported in less than 10% of cases in the literature) and even more rarely with non-dissecting distal aortic or peripheral aneurysms [13,14,15,16]. Consequently, due to the greater familiarity of clinicians with Marfan syndrome, LDS patients who initially present with aortic root aneurysms, along with systemic characteristics commonly seen in Marfan syndrome, are at risk of being misdiagnosed as having Marfan syndrome [11]. This is a critical issue since LDS patients, especially those of types 1 and 2, often experience aortic dissection at aortic diameters below the intervention thresholds applied to Marfan syndrome patients [17]. In addition, recently, the case of a 46-year-old woman affected by LDS4 presenting ectopia lentis was reported, underscoring the overlapping features between LDS4 and Marfan syndrome and the challenges associated with making an accurate diagnosis [18].

While some clinical features in LDS4 are described as extremely rare, the continuous discovery of new case reports suggests that such assumptions might be premature. For example, developmental delay has recently been documented in a French family with LDS4 [19]. Furthermore, while the spectrum of clinical features associated with LDS4 is broad, the genotype-phenotype correlations are not yet fully elucidated. Thus, it is important to document in detail new cases of LDS4 who undergo genetic testing in order to better comprehend the clinical spectrum of each pathogenic variant.

In this report, we present the case of a 61-year-old man who was previously misdiagnosed with Marfan syndrome but was revealed by genetic analysis to have LDS4. The initial diagnosis was set clinically, even though the patient did not meet the necessary revised Ghent criteria. We performed a molecular modeling study to correlate the genotype to phenotype and observed that the determined missense pathogenic variant has devastating effects on the protein structure level.

## 2. Materials and Methods

### 2.1. Description of Patient

**Figure 1:**
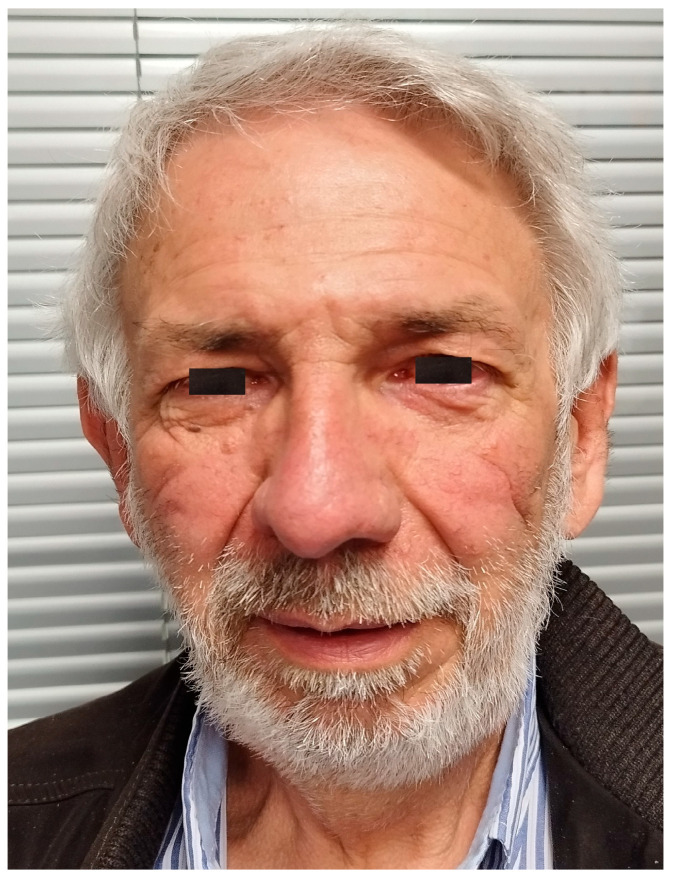
Image of the patient.

**Figure 2:**
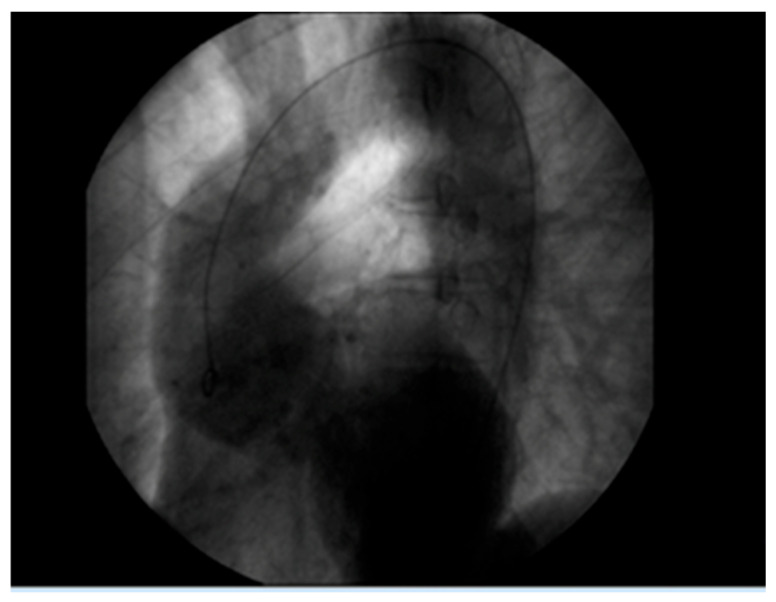
Aortic root aneurysm of 7 cm visualized on coronography.

**Figure 3:**
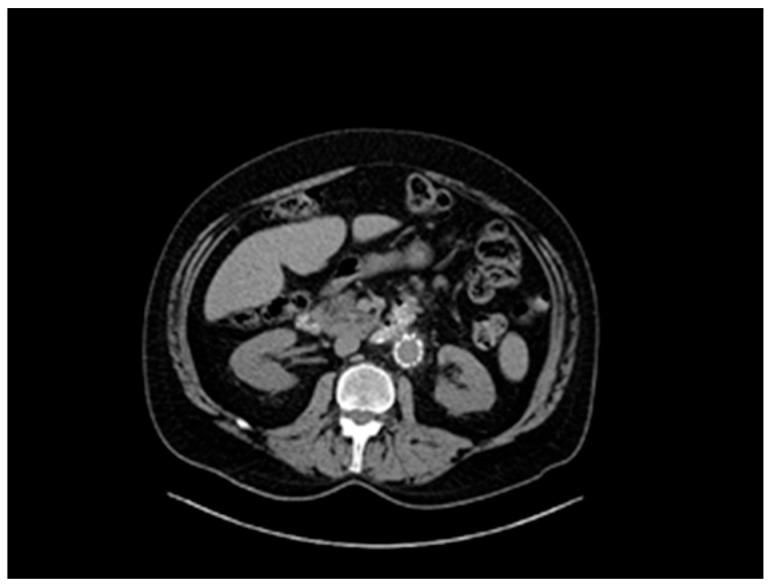
Abdominal aorta stent graft.

A 61-year-old patient with a history of probable Marfan syndrome was examined after referral from the treating physician because of hyperthyroidism. The tentative diagnosis of Marfan syndrome was set two decades ago based on the patient’s phenotype and a personal record of a Sinus of Valsalva aneurysm. The phenotype included a tall and lean statue, high-arched palate, crowded teeth, arachnodactyly, and divergent strabismus (Figure 1). The patient gave informed consent for the publication of his photo. The patient’s height was 188 cm, and his current weight was 88 kg. At the age of 49, a diagnosis of a 7 cm Sinus of Valsalva aneurysm was made (Figure 2), accompanied by concurrent grade 3 to 4 aortic valve insufficiency. The aortic aneurysm’s Z-score was 7.6, considering the patient’s sex, age, height, and weight. Previous surgical intervention involved an aortic valve replacement. Six years later, at the age of 55, the patient was hospitalized for an infrarenal abdominal aorta aneurysm measuring 4.9 cm, which was managed with endovascular repair and stent placement (Figure 3). Other medical history included an ischemic stroke in the right middle cerebral artery at age 56, attributed to insufficient anticoagulation in the context of a prosthetic aortic valve. The stroke did not result in any lasting neurological deficits. The patient did not present with cerebral aneurysms and had no history of arterial hypertension, hypercholesterolemia, diabetes mellitus, or active smoking.

### 2.2. Genetic Counseling

**Figure 4:**
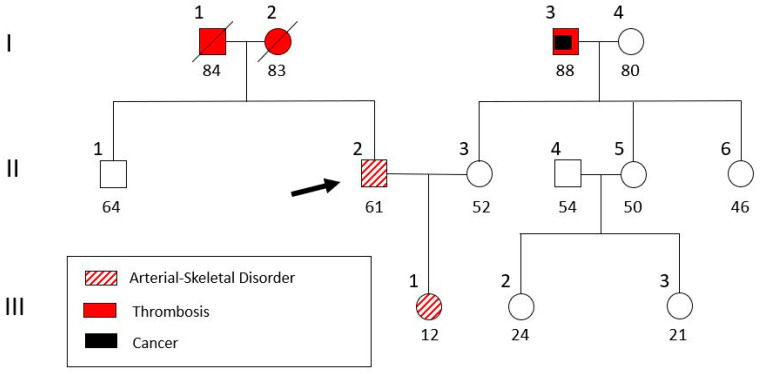
Pedigree of the patient’s family. The patient (II-2, indicated with an arrow) and his daughter (III-1) were genetically analyzed.

The discovery of an aortic aneurysm in a location other than the aortic root raised doubts about the initial diagnosis of Marfan syndrome, and the patient was referred to a clinical geneticist. The family history taken (Figure 4) revealed that the patient (II-2) has a daughter (III-1) with a similar phenotype (tall and lean stature, a high-arched palate, crowded teeth, and arachnodactyly) and, furthermore, debutant scoliosis and divergent strabismus. The patient’s parents (I-1 and I-2) both had a history of non-specified thrombosis (Figure 4). After genetic counseling and informed consent, a blood sample was extracted from the patient for genomic DNA analysis. The study was approved (RPURI9002) by the Bioethics Committee of the University Research Institute of Maternal and Child Health and Precision Medicine at the School of Medicine of the National Kapodistrian University of Athens.

### 2.3. Genetic Analysis

Total DNA isolation was performed using a Nucleospin^®^ Blood Quickpure kit (Macherey Nagel GmbH, Düren, Germany), following the guidelines of the manufacturer. Subsequently, whole exome sequencing (WES) was performed with the patient’s DNA for identification of the pathogenic variant using a Novaseq 6000 (Illumina, San Diego, CA, USA). This was followed by a bioinformatic study of the obtained DNA sequence and a comparison with a reference sequence (GRCh37). The variation analysis was performed using the VarSome Clinical platform and varAFT 2.14 (http://varaft.eu), which uses genotype-phenotype correlation predictions from several genetic databases. The impact of point missense changes on protein structure and function is checked using the consensus prediction algorithm, which combines and takes into account the prediction of 10 prediction algorithms: SIFT, PolyPhen-2 HDIV, PolyPhen2 HVAR, GERP++, Mutation Taster, Mutation Assessor, FATHMM, LRT, SiPhy, and PhyloP, calculating a score ranging from −2 to 3. The findings were categorized based on guiding criteria developed by the American College of Medical Genetics and Genomics and the Association for Molecular Pathology [20]. For confirmation, targeted DNA sequencing of the *TGFB2* gene region containing the pathogenic variant was carried out in the patient’s and daughter’s DNA samples, using an automated capillary sequencer ABI 3730 XL Analyzer (Applied Biosystems, Waltham, MA, USA).

### 2.4. Molecular Modeling

Homology protein modeling was performed using the Molecular Operating Environment (MOE 2020.09)) software package (Chemical Computing Group, Montreal, QC, Canada). The three-dimensional structure of the target protein TGF-β was predicted based on its amino acid sequence homology to experimentally determined structures of related proteins, referred to as templates. Herein, the 3D structure of pro-TGF-β 1 (*Sus scrofa*) with PDB id: 3RJR was used. First, sequence alignment was conducted between the target protein and selected template structures using MOE’s sequence alignment tools. Templates were chosen based on significant sequence identity and structural similarity to the target protein. Subsequently, the homology modeling module in MOE was utilized to generate a preliminary 3D model of the target protein. This involved constructing a model that mimicked the structure of the templates while incorporating the unique features of the target protein sequence. The quality of the homology model was evaluated using MOE’s built-in validation tools, including assessment of steric clashes, evaluation of model geometry, and estimation of reliability based on sequence identity and template/template-target alignment. To refine the homology model, energy minimization, side-chain optimization, and loop modeling were performed using MOE’s refinement and optimization tools.

### 2.5. Molecular Dynamic Simulations

The validity of the homology model was assessed through additional computational techniques, such as molecular dynamics simulations. Molecular dynamics (MD) simulations are invaluable for deciphering the structural alterations induced by pathogenic/likely pathogenic variants in proteins. MD simulations use classical mechanics to model the motion of atoms and molecules over time. Starting from an initial configuration, the positions and velocities of atoms are updated at each time step by solving Newton’s equations of motion. These calculations rely on force fields, mathematical models that describe the potential energy of the system based on atomic interactions. The simulation begins with system preparation, including solvation and neutralization, followed by energy minimization to remove unfavorable interactions. The system is then equilibrated to reach a stable state under desired temperature and pressure conditions. Finally, the production phase of the simulation generates trajectories that capture atomic motions over time. The system was energy-minimized using the appropriate force field parameters implemented in MOE to remove steric clashes and relax the structure. Subsequently, the system was equilibrated to achieve stable temperature and pressure conditions using MD simulations with position restraints on the protein atoms. Following equilibration, production MD simulations were performed to sample the conformational space of the protein over time. A suitable integration time step and simulation length were chosen based on system size and computational resources. During the MD simulations, MOE’s analysis tools were utilized to monitor various structural properties, including root-mean-square deviation (RMSD), root-mean-square fluctuation (RMSF), and secondary structure evolution. MDS took place in an SPC water-solvated, periodic environment. Water molecules were added using the truncated octahedron box extending 7 Å from each atom. The molecular systems were neutralized with counter-ions as required. For this study, all MDSs were performed using the NVT ensemble in a canonical environment at 300 K and a step size equal to 2 fs for a total 10 ns simulation time. The investigation of structural integrity using MD simulations is an indispensable approach in modern science. By simulating molecular systems under various conditions, researchers can uncover fundamental principles governing stability and functionality. This knowledge not only enhances our understanding of molecular systems but also drives innovations in medicine, materials, and biotechnology.

## 3. Results

**Figure 5:**
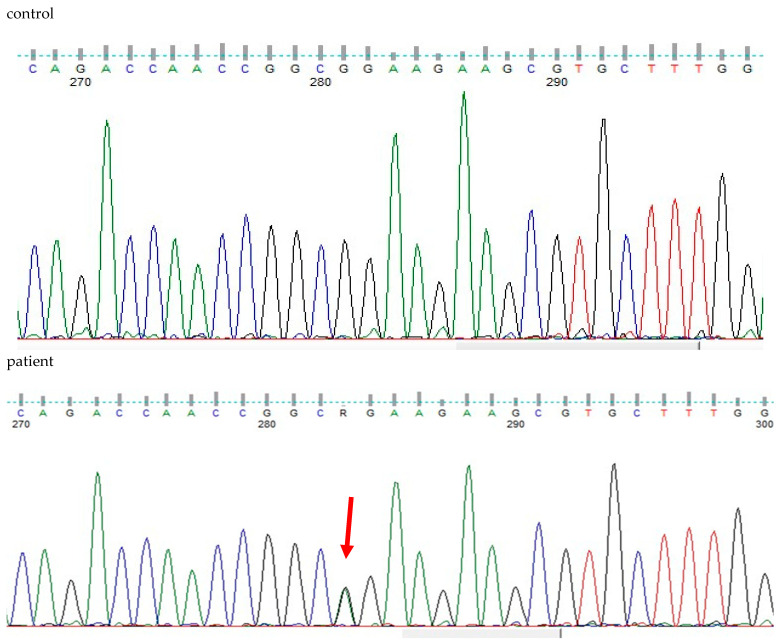
DNA sequencing findings of exon 5 of the heterozygous patient’s *TGFB2* gene (NM_003238.6(TGFB2):c.896G>A (p.Arg299Gln) in comparison to a healthy control.

WES analysis of thousands of genes (including *TGFB2*, *TGFB3*, *TGFBR1*, *IPO8*, *SMAD2*, and *SMAD3*) detected a deleterious heterozygous missense pathogenic variant NM_003238.6 (TGFB2):c.896G>A (p.Arg299Gln). Sanger sequencing confirmed the finding (Figure 5), and the diagnosis of Loeys-Dietz syndrome type 4 (LDS4) was established for the patient, as the disorder is apparently caused by a de novo dominant pathogenic variant in the *TGFB2* gene. The patient’s daughter was also a heterozygote for the observed pathogenic variant, which is pathogenic (ƒ = 0.000000684) and has been previously detected in another LDS4 patient [21].

The clinical features of our patient and his daughter are described in Table 1. After assessment by a clinical genetics expert, no additional LDS4 clinical manifestations, such as striae, myopia, cataract, scoliosis, kyphosis, flat foot, or chest deformity, were detected. Furthermore, recent imaging tests of the patient revealed no additional LDS-related vascular manifestations, such as vascular tortuosity or ectasia.

**Figure 6:**
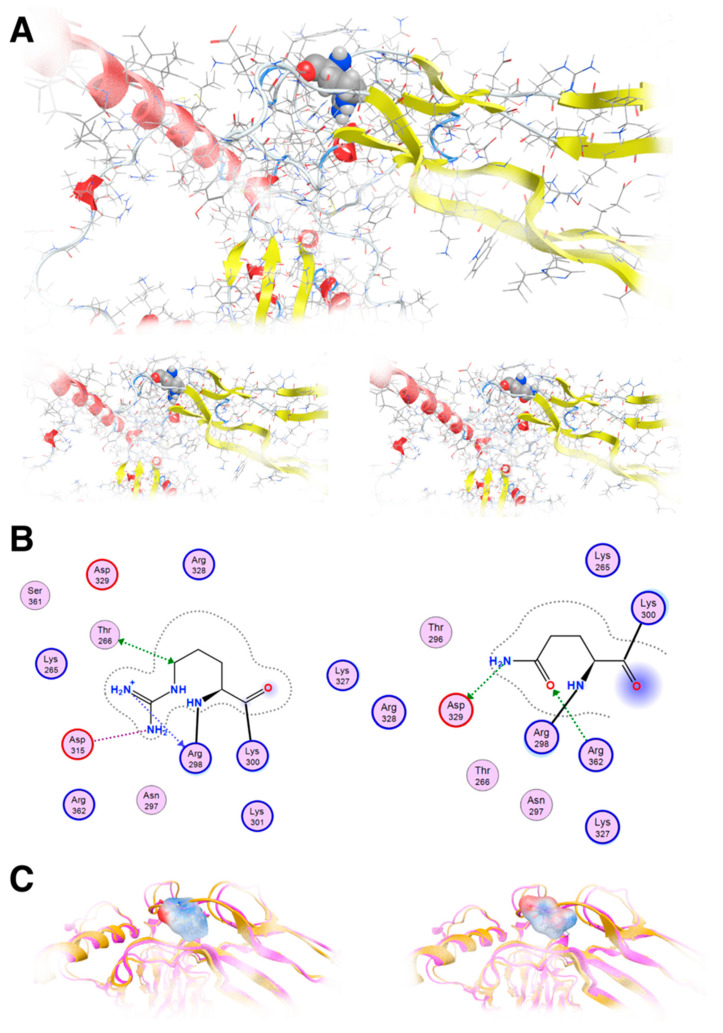
Molecular modeling of the mutant TGFB2 R299Q model. (**A**): Upper: The template used to show R299 in a ball and stick representation; Lower: The template and the designed model of the TGFB2 R299Q (left and right, respectively). (**B**): Molecular interaction of two diagrams of the R299 and the Q299, respectively. Protein residues appear in spheres (red and blue circled for acidic and basic respectively). (**C**): Electrostatic cloud surface calculation for both R and Q (left and right, respectively).

Our molecular modeling study highlighted the fact that the change of the arginine (Arg) to a glutamine (Gln) residue indeed had significant effects on the structure and function of the protein, as the location of these residues is involved in critical interactions within the protein. Arginine and glutamine have different chemical properties. Arginine is a positively charged amino acid with a long, flexible side chain, while glutamine is uncharged with a shorter side chain. When an arginine residue that plays a crucial role in stabilizing the protein structure or in binding to other molecules is changed to glutamine, it can disrupt these interactions and alter the protein’s three-dimensional structure (Figure 6A). The wild-type of arginine residue is involved in forming salt bridges or hydrogen bonds with nearby amino acids or other molecules; substituting it with glutamine, which lacks the positive charge of arginine, would weaken or abolish these interactions. This disruption can lead to misfolding of the protein or loss of its functional properties. The arginine residue interacts via metal/ion contact with Asp315 and Thr268 via hydrogen bonding. The glutamine residue lost both of these vital interactions; on the contrary, it established hydrogen bonds to Asp329 and Arg362 because its side chain is shorter than arginine’s side chain (Figure 6B). Moreover, the vital positive charges of the arginine residue have migrated and were significantly weakened by the introduction of the glutamine residue, as can be deduced by the electrostatic cloud representations for these two residues in the proximity of the protein (Figure 6C).

**Figure 7:**
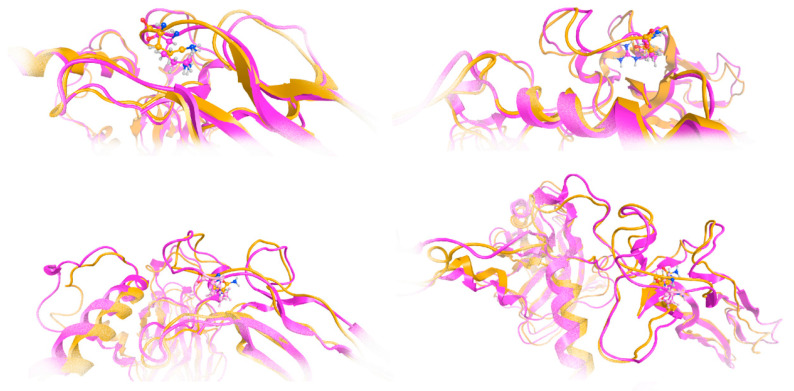
Investigation of structural integrity upon molecular dynamics simulations. Molecular dynamics (MD) simulations are a powerful computational tool used to explore the behavior and properties of molecular systems over time. One of the key applications of MD simulations is the investigation of structural integrity, which involves studying the stability and resilience of molecular structures under various conditions. This approach provides valuable insights into the physical and chemical characteristics of molecules, especially in fields such as biochemistry, materials science, and drug discovery. The model of the mutant TGFB2 299Q (magenta ribbon) was superposed to the final model upon MDs in various poses/viewpoints (orange ribbon).

**Figure 8:**
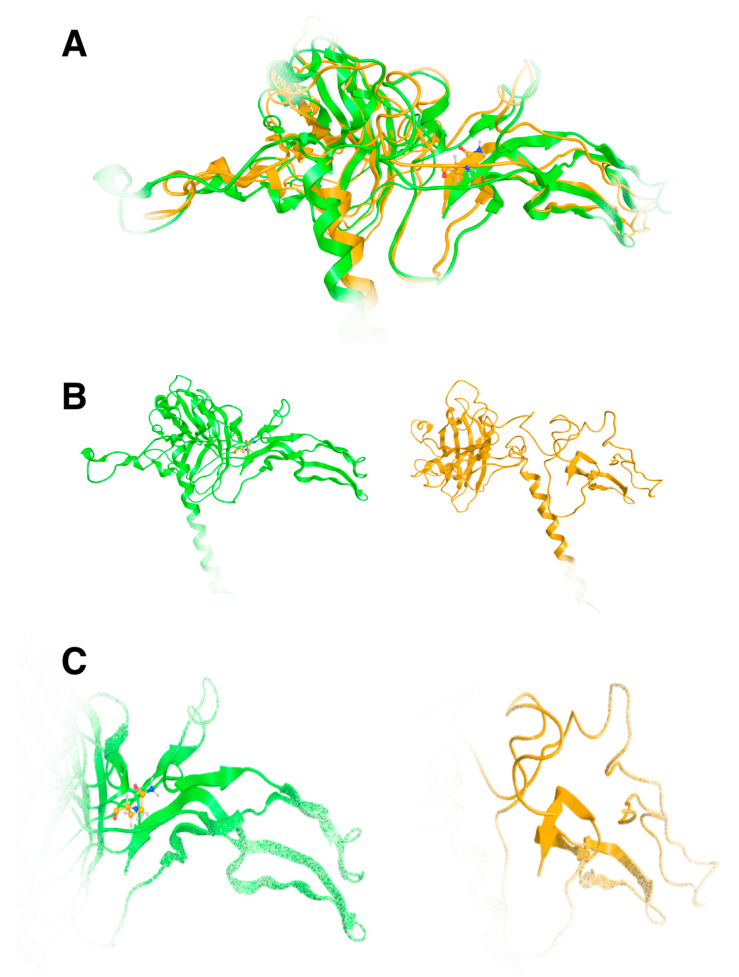
Superposition of the wild-type TGFB2 (green ribbon) and the model of the 299Q mutated protein upon MDs. It is evident that a significant amount of the protein has been lost, which is indicative of biological loss of function. (**A**): Superposed models, (**B**): Wild and mutated 3D models of TGFB2, (**C**): Same as B, but close up on the binding site.

The effect of the arginine (R) to glutamine (Q) variant was investigated by exhaustive molecular dynamics simulations. It was shown that the structure of the mutated protein changes significantly upon MDs (Figure 7). Structural integrity refers to the ability of a molecule or system to maintain its structure and function under different environmental or physical stresses. For biological molecules such as proteins, DNA, or membranes, structural integrity is crucial for their proper function. Any loss of structural stability can lead to dysfunction, which is often associated with diseases. Similarly, for synthetic materials or nanostructures, maintaining structural integrity is essential for their performance and reliability in applications. By simulating the motions of atoms over time, MD can elucidate how pathogenic variants influence the overall conformation, folding, dynamics, and interactions of proteins. These simulations reveal changes in secondary and tertiary structures, alterations in dynamic behavior and flexibility, and modifications in intra-protein and protein-ligand interactions. Moreover, MD simulations provide insights into how pathogenic variants affect binding affinities and water dynamics within the protein’s environment. Wild-type and mutant protein dynamics comparison in molecular dynamics simulations involves studying the differences in behavior between the natural (wild-type) version of a protein and its altered (mutant) form. By simulating both versions, researchers can analyze how pathogenic variants affect the stability, flexibility, and overall structural integrity of the protein. These comparisons often reveal changes in folding, intermolecular interactions, or regions of increased flexibility in the mutant protein. Such insights are critical for understanding the functional consequences of pathogenic variants, especially in the context of diseases, and can guide drug design or protein engineering efforts (Figure 7 and Figure 8). The two structures were then superposed, and it became obvious that there was quite a lot of conformational rearrangement in the mutated protein (Figure 8A). Interestingly, a series of b-sheets was completely lost in the mutated protein (Figure 8B,C). This is a clear effect of the pathogenic variant that resulted in a conformational change, structural compromise, and, thus, loss of function. Substituting the arginine with glutamine indeed impacted the three-dimensional structure of the protein, potentially affecting its function and leading to various biological consequences.

The charge difference between the two amino acids is crucial. Arginine has a positively charged guanidinium group at physiological pH, which is important for interactions with negatively charged molecules such as phosphate groups of nucleic acids, acidic residues in proteins, or membrane phospholipids. Glutamine, on the other hand, is neutral at physiological pH. The loss of positive charge can disrupt electrostatic interactions that are crucial for the protein’s stability, folding, or function. Moreover, arginine’s side chain can form multiple hydrogen bonds due to the presence of the guanidinium group, which is important for stabilizing protein structures and interacting with other molecules. On the other hand, glutamine’s side chain contains an amide group that can also form hydrogen bonds, but it cannot form as many as arginine can. It is of uttermost importance to protein 3D structure that arginine residues are used for maintaining structural integrity through salt bridges or hydrogen bonds.

In the Arg299Gln case, replacing arginine with glutamine clearly leads to destabilization and conformational changes in 3D folding. Lastly, another issue that merits to be mentioned is the additional possibility of epigenetics and post-translational modifications. While arginine residues can be subjected to post-translational modifications such as methylation, this may not be possible with glutamine, potentially affecting regulation and function on the epigenetic level.

## 4. Discussion

LDS4 exhibits clinical features similar to those of Marfan syndrome, though each condition retains distinct characteristics. Table 2 highlights the shared and unique features of these syndromes. Our patient exhibited several clinical features commonly associated with LDS4, as identified in a recent systematic review [3]. The most prominent feature observed is an aortic root aneurysm, which occurs in 61% of LDS4 cases. Additionally, 28% of patients exhibit aneurysms in other vessels, excluding the aortic root, subclavian artery, or cerebral vessels. High-arched palate is another feature seen in 28% of LDS4 patients, while arachnodactyly is present in 22%. Exotropia, affecting 17% of patients, and crowded teeth, which were not observed in any of the previously described LDS4 cases, further distinguish our patient’s clinical presentation [3]. Our patient’s pathogenic variant has been previously detected in another LDS4 patient [22]. In that reported case, the individual exhibited marfanoid characteristics, such as arachnodactyly and a high palate, along with a thoracic aortic aneurysm measuring 4.1 cm, similar to our patient’s condition. However, the other case presented hypertelorism and retrognathia, features that were not observed in our patient [22].

All pathogenic variants causing LDS interfere with the TGF-β cascade, leading to increased TGF-β activity [21]. The TGF-β superfamily consists of multifunctional cytokines that serve as ligands for cell surface receptors and play a pivotal role in numerous critical processes during regular cellular growth and development [21]. The interaction between TGF-βs and their receptors triggers a wide range of cellular reactions, leading to the phosphorylation of Smad proteins. Once phosphorylated, Smad proteins migrate to the nucleus, where they regulate the transcription of specific target genes [21]. TGF-β is integral to embryonic development, particularly in the shaping of craniofacial structures, and pathogenic variants in the genes responsible for encoding these isoforms can give rise to a range of orofacial and craniofacial abnormalities [23]. For instance, pathogenic variants in the TGFB1 and TGFB3 genes are linked to the development of cleft lip and cleft palate, conditions that are frequently observed in individuals with LDS [23]. Our molecular modeling analysis revealed the devastating effect of the pathogenic variant at the 3D structural level, as it resulted in a conformational change and structural compromise, resulting in a loss of function of TGF-β2. Substituting the arginine with glutamine, two amino acids with very different biochemical properties, indeed impacted the three-dimensional structure of the protein, leading to the clinical phenotype of LDS4.

Most of the documented cases of LDS4 in the literature involve patients exhibiting typical craniofacial and skeletal abnormalities alongside aortic aneurysms, with or without vascular dissection, similar to our patient’s presentation. Genetic analysis of these cases has consistently identified missense and loss-of-function variants in the *TGFB2* gene [6,7,22]. However, a study by Ritelli et al. reported an Italian family presenting systemic features such as a high palate, hypoplastic uvula, joint hypermobility, and scoliosis, along with tortuosity and ectasia of cerebral, carotid, and vertebral arteries, without arterial aneurysm or dissection [24]. Genetic analysis of this family revealed a loss-of-function splice site variant, c.839-1G>A, in the *TGFB2* gene, indicating that not all loss-of-function variants of this gene result in severe vascular abnormalities [24]. Additionally, Gago-Díaz et al. described two members of a Spanish family presenting with aortic dilation, aortic dissection, and bicuspid aortic valve [8]. Apart from minor connective tissue abnormalities observed in some family members, characteristic systemic features of LDS or other connective tissue disorders were absent [8]. Genetic testing identified a missense pathogenic variant, c.C1042T: p.R348C, in the *TGFB2* gene, indicating functional alterations in the TGFβ-2 protein and suggesting that *TGFB2* gene pathogenic variants can lead to non-syndromic aortic disease [8]. Table 3 presents all missense variants in the *TGFB2* gene that are reportedly associated with LDS4 cases.

As the number of individuals diagnosed with LDS increases, it is important to advance our knowledge of the spectrum of medical features of each subtype. This knowledge is instrumental in refining management approaches and enhancing recommendations for follow-up care. It is essential to medical practice, genetics, and basic research to provide insights from multi-disciplinary approaches in an effort to functionally characterize the clinical phenotype. Our genetic analysis identified a previously reported TGFB2 missense pathogenic variant, with distinct clinical presentations observed between our patient and the one described by Renard et al. [22]. This underscores the unique contribution of our findings in expanding the understanding of genotype-phenotype correlations and the diverse clinical spectrum associated with TGFB2 variants. Furthermore, there is currently no cure for LDS, and treatment is limited to symptomatic management, primarily through surgical interventions. These interventions carry inherent risks for the patient and impose a significant financial burden on healthcare systems. Further elucidation of the pathogenic mechanisms at the molecular level could enable the development of targeted therapies, offering more effective and personalized treatments and ultimately improving the quality of life for affected patients.

## Figures and Tables

**Table 1:** Clinical features of our patient and his daughter.

Clinical Features of the Patient	Clinical Features of the Daughter
Sinus of Valsalva aneurysm (7 cm)	-
Infrarenal abdominal aorta aneurysm (4.9 cm)	-
High-arched palate	High-arched palate
Arachnodactyly	Arachnodactyly
Exotropia	Exotropia
Crowded teeth	Crowded teeth

**Table 2:** Common and distinct features of Marfan syndrome and LDS4.

Feature	Marfan Syndrome	LDS4
**Cardiovascular Features**		
Aortic root aneurysm	+	+
Thoracic aorta dissection	+	+
Arterial tortuosity		+
Bicuspid aortic valve	+	+
**Skeletal Features**		
Tall stature	+	+
Arachnodactyly	+	+
Clubfoot		+
Scoliosis	+	+
Pectus deformities	+	+
**Orofacial Features**		
Hypertelorism		+
Bifid uvula		+
Cleft palate		+
**Ocular Features**		
Ectopia lentis	+	
**Skin Features**		
Skin striae	+	+

**Table 3:** Missense variants in the *TGFB2* gene reportedly associated with LDS4 cases.

Codon Number	Codon Change	Amino-Acid Change	Reference
79	CAG-CTG	Gln-Leu	Schepers et al., 2018 [25]
82	GCG-TCG	Ala-Ser	Schepers et al., 2018 [25]
153	CGT-CAT	Arg-His	Posey et al., 2017 [26]
298	CGG-CAG	Arg-Gln	Stengl et al., 2020 [27]
299	CGG-CAG	Arg-Gln	Renard et al., 2012 [22]—Present case
299	CGG-TGG	Arg-Trp	Lindsay et al., 2012 [7]
302	CGT-CCT	Arg-Pro	Trujillano et al., 2017 [28]
320	CGT-TGT	Arg-Cys	Gago-Díaz et al., 2014 [8]
338	CCC-CAC	Pro-His	Lindsay et al., 2012 [7]
384	GAT-CAT	Asp-His	Schepers et al., 2018 [25]
413	TGC-TGG	Cys-Trp	Stengl et al., 2020 [27]

## Data Availability

All related data and materials are available.
